# In Silico and In Vivo Analysis of Amino Acid Substitutions That Cause Laminopathies

**DOI:** 10.3390/ijms222011226

**Published:** 2021-10-18

**Authors:** Benjamin E. Hinz, Sydney G. Walker, Austin Xiong, Rose A. Gogal, Michael J. Schnieders, Lori L. Wallrath

**Affiliations:** 1 Department of Biochemistry, University of Iowa, Iowa City, IA 52242, USA; benjamin-hinz@uiowa.edu (B.E.H.); sydney-walker@uiowa.edu (S.G.W.); axiong23@amherst.edu (A.X.); michael-schnieders@uiowa.edu (M.J.S.); 2 Department of Biomedical Engineering, University of Iowa, Iowa City, IA 52242, USA; rose-gogal@uiowa.edu

**Keywords:** congenital muscular dystrophy, Emery–Dreifuss muscular dystrophy, intermediate filaments, laminopathy, lamins, nuclear envelope

## Abstract

Mutations in the *LMNA* gene cause diseases called laminopathies. *LMNA* encodes lamins A and C, intermediate filaments with multiple roles at the nuclear envelope. *LMNA* mutations are frequently single base changes that cause diverse disease phenotypes affecting muscles, nerves, and fat. Disease-associated amino acid substitutions were mapped in silico onto three-dimensional structures of lamin A/C, revealing no apparent genotype–phenotype connections. In silico analyses revealed that seven of nine predicted partner protein binding pockets in the Ig-like fold domain correspond to sites of disease-associated amino acid substitutions. Different amino acid substitutions at the same position within lamin A/C cause distinct diseases, raising the question of whether the nature of the amino acid replacement or genetic background differences contribute to disease phenotypes. Substitutions at R249 in the rod domain cause muscular dystrophies with varying severity. To address this variability, we modeled R249Q and R249W in Drosophila *Lamin C*, an orthologue of *LMNA*. Larval body wall muscles expressing mutant *Lamin C* caused abnormal nuclear morphology and premature death. When expressed in indirect flight muscles, R249W caused a greater number of adults with wing posturing defects than R249Q, consistent with observations that R249W and R249Q cause distinct muscular dystrophies, with R249W more severe. In this case, the nature of the amino acid replacement appears to dictate muscle disease severity. Together, our findings illustrate the utility of Drosophila for predicting muscle disease severity and pathogenicity of variants of unknown significance.

## 1. Introduction

Mutations in the human *LMNA* gene cause a wide variety of diseases with diverse symptoms. These diseases include muscular dystrophy, familial partial lipodystrophy, neuropathy, cardiac disease, and premature aging syndromes, which are collectively referred to as laminopathies [1,2]. Affected individuals often exhibit symptoms of more than one disease, suggesting that the laminopathies represent a continuum. This often complicates clinical diagnoses.

The *LMNA* gene encodes the A-type lamins, lamins A and C, which are produced by alternative splicing [3,4]. In contrast, there are two B-type lamins, lamin B1 and B2, which are encoded by separate genes *LMNB1* and *LMNB2* [5,6]. The A- and B-type lamins are intermediate filaments with a conserved domain structure consisting of a head, rod, and tail that includes an Ig-like fold [7,8]. Dimerization occurs through the rod domain [9]. Lamins interact in a head to tail manner to form a polymer and the polymers interact laterally to form an irregular meshwork that lines the inner side of the nuclear envelope [10,11,12,13]. The A- and B-type lamins assemble into independent networks that line the inside of the inner nuclear membrane [14]. Despite A- and B-type lamins having similar structure and function, these networks differ in their assembly and organization [15]. In addition, mutant A- and B-type lamins give rise to clinically distinct diseases in humans [16] and distinct defects in model organisms [17,18].

Lamins serve multiple functions in the cell. They provide structural support for the nucleus and ensure the integrity of the nuclear envelope [19,20]. Support for this statement comes from studies showing that transient nuclear rupture and increased DNA damage are observed in skeletal muscle nuclei, which experience mechanical stress [21,22]. Lamins provide a scaffold for attachment of chromatin to the nuclear envelope at specific genomic locations called lamin-associated domains (LADs), which play a role in the orchestration of proper gene expression [19,23,24,25]. LADs attract specific non-histone chromosomal proteins and are enriched for histone H3K9 tri-methylation, an epigenetic gene silencing mark [23]. Lamins also interact with many other nuclear envelope proteins, including nuclear pore proteins [26]. Lamins are necessary for uniform distribution of nuclear pores throughout the nuclear envelope [18,27]. In addition, lamins interact with SUN domain proteins, which are components of the Linker of Nucleoskeleton and Cytoskeleton (LINC) complex that span the nuclear envelope [26,28,29]. This interaction is critical for mechanotransduction, a process that translates mechanical signals from the cytoskeleton to biochemical responses inside the nucleus [21,30,31]. Lamins also bind the retinoblastoma protein (Rb) and regulate cell cycle progression [28]. Thus, lamins serve as a scaffold for the structure of the nucleus and participate in nuclear-cytoskeletal connections and signaling.

Given that lamins are expressed in nearly all cell types, it is unclear how mutations in *LMNA* cause tissue-restricted disease phenotypes [16,29,30,31]. Several non-mutually exclusive hypotheses have been proposed to explain this tissue-specific paradox. One hypothesis posits that lamins weaken the nuclear envelope, causing susceptibility to mechanical stress [32]. This could explain the skeletal muscle and cardiac laminopathies, but perhaps not adipose tissue disorders. For tissues not under mechanical stress, the pathogenic mechanisms might relate to the function of lamins in genome organization, with abnormalities in the lamina causing altered gene expression [23,32]. Alternatively, tissue-specific defects could arise from cell type-specific interactions between lamins and nuclear envelope proteins [31]. Last, another hypothesis posits that mutant lamins cause defective stem cell regeneration, consistent with the fact that adult tissues that undergo self-renewal and/or repair are affected in disease [33]. This hypothesis could also explain the relatively late onset of many laminopathies.

To date, several hundred mutations in *LMNA* have been identified (http://www.umd.be/LMNA/ accessed on 1 June 2020; https://databases.lovd.nl/shared/varants/LMNA/unique accessed on 1 June 2020) Most of these mutations are single base changes that lead to amino acid substitutions; however, mutations that alter splicing have also been discovered. The amino acid substitutions map throughout the lamin protein [34]. Here, we in silico map several hundred amino acid substitutions onto previously determined structures of portions of lamin A/C to examine genotype-phenotype relationships.

Amino acid substitutions at different positions within A-type lamins can cause the same disease phenotypes [35,36,37]. In addition, the same amino acid substitution in lamin A/C can result in different disease phenotypes, even among closely related family members [38,39]. It is also true that different amino acid substitutions at the same position within lamin A/C can cause clinically distinct diseases [35,40]. Collectively, these findings strongly suggest that genetic background plays a role in the disease phenotypes manifested in affected individuals. To analyze the contributions of specific amino acid substitutions to laminopathy phenotypes we modeled specific disease-causing mutations in *Drosophila melanogaster*. This allowed us to determine if different amino acid substitutions at the same position caused similar defects in a defined genetic background.

## 2. Results

### 2.1. The Position and Predicted Solvent Accessibility of an Amino Acid Substitution Do Not Correspond to Disease Phenotypes

**Figure 1:**
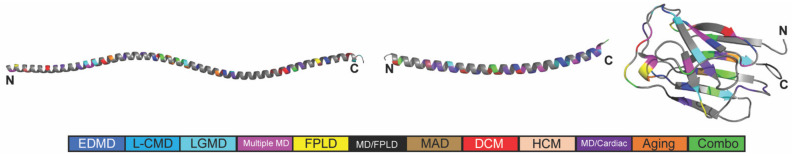
Disease-associated amino acids were in silico mapped onto determined structures for lamin A/C. Previously determined structures of portions of lamin A/C include coil 1B (PDB: 6SNZ, residues 63–222) and coil 2B (PDB: 1X8Y, residues 313–385) of the rod domain, and the Ig-like fold (PDB: 1IVT, residues 428–549). Colored residues indicate the position of amino acid substitutions that cause laminopathies (color code bar below).

To determine the relationship between the position of amino acid substitutions and specific diseases, the amino acids altered in disease were mapped onto determined structures of lamin A/C (Figure 1). Three-dimensional structural information was available for three portions of lamin A/C: two regions of the rod domain (PDB: 6SNZ; 1X8Y) and the Ig-like fold (PDB: 1IVT). In total, 315 amino acid substitutions across 205 residues were mapped to these structures. Each disease was given a color code. When amino acid substitutions at a specific position resulted in multiple diseases, a different color code was designated. The mapping confirmed prior studies performed with a smaller dataset, showing mutations that affect all three domains can result in the same disease [35,36,37]. For example, substitutions causing autosomal dominant Emery-Dreifuss muscular dystrophy (AD-EDMD) are throughout all three domains used (Figure 1). It was also apparent that different amino acid substitutions at the same position can cause different diseases. Such findings suggest that properties of amino acid themselves might be determinants of disease phenotypes.

The solvent accessibility of amino acids in folded protein structures is a property that influences their function. Prior studies suggested that amino acid substitutions in the lamin A/C Ig-like fold domain predicted to be solvent-inaccessible (i.e., buried in the interior) were more likely to cause skeletal muscular dystrophy [41]. By contrast, amino acid substitutions in the lamin A/C Ig-like fold domain predicted to be solvent-accessible (i.e., on the exterior) were more likely to cause lipodystrophy. With an expanded clinical dataset now available, we revisited this relationship. To do so, we submitted the Ig-like fold structure (PDB: 1IVT) to the GETAREA 1.0 online software, which calculates Surface-Accessible Solvent Area (SASA) scores for each amino acid residue [42]. The results showed that 18 of 35 (51%) amino acids within the Ig-like fold domain affected in cases of muscular dystrophy were predicted to be solvent accessible (Supplementary Table S1). Eight of the 12 (67%) amino acids within the Ig-like fold domain that cause lipodystrophy were predicted to be solvent accessible. There was no statistically significant difference between these proportions (*p* = 0.3314). Thus, amino acid substitutions resulting in either muscular dystrophy or lipodystrophy are found at relatively similar percentages in the interior and on the exterior of the Ig-like fold domain, strongly suggesting that solvent accessibility in the Ig-like fold domain is not a determinant of disease phenotypes.

### 2.2. In Silico Predicted Binding Pockets of the Ig-like Fold Map to Disease-Causing Amino Acid Substitutions

**Figure 2:**
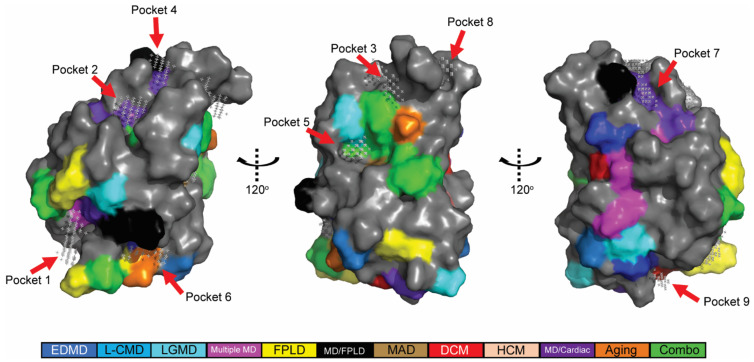
Predicted protein binding pockets in the Ig-like fold domain map to positions of disease-related amino acid substitutions. A surface model of the Ig-like fold domain (PDB: 1IVT; gray) is shown with the position of disease-related amino acid substitutions indicated by colors (color code bar below). Protein partner binding pockets (white hashes) were predicted by POCASA v1.1 [43]. 7/9 are overlaid on the surface model.

Given that the position of amino acid substitutions within lamin A/C did not appear to correspond to particular disease phenotypes, we wondered if specific amino acid substitutions disrupted interactions with partner proteins. Using POCASA v1.1 [43], nine potential binding pockets were predicted on a surface structure of the Ig-like fold (Figure 2). Of the nine predicted binding pockets, seven overlapped with surface-exposed amino acid residues associated with disease (Figure 2, Supplementary Table S2). Disease causing amino acid substitutions within these seven pockets disrupted the interaction of experimentally determined binding partners [44] (Supplementary Table S2).

### 2.3. Generation of an In Silico Full-Length Human Lamin A/C

**Figure 3:**
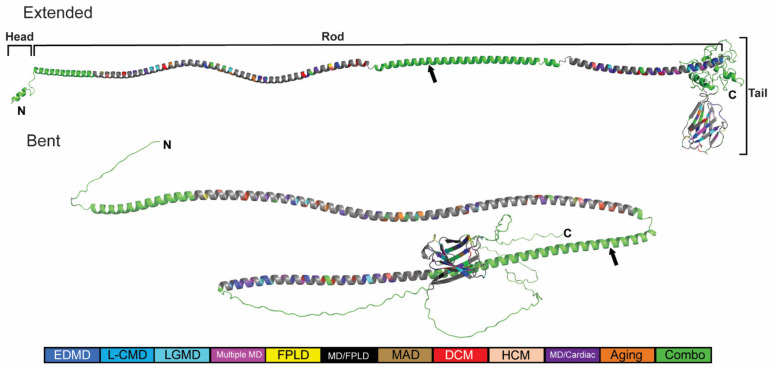
In silico predicted full-length models were generated for lamin A/C. Deep learning based predictive full-length model of a lamin A/C monomer generated by trRosetta [45] (green) in which the previously determined structures were spliced into the model (gray with color-coded amino acids). trRosetta predicted an “extended” lamin A/C monomer (top). Full-length lamin A/C model was generated by AlphaFold v2.0 and disease-associated residues were color-coded [49]. The top four out of five predicted models possessed a bent conformation (bottom).

To visualize where these disease-causing amino acids were positioned in lamin A/C, we generated a full-length in silico model using trRosetta [45] (Figure 3, top). We started with the three portions of lamin A/C that had known structures (PDB: 6SNZ; 1X8Y; 1IVT) and optimized them using Amber [46]. An initial full-length structure was predicted from sequence using trRosetta [45]. We spliced the optimized homology structures into the predicted full-length model. Finally, we optimized the full-length prediction of the lamin A/C structure using the AMOEBA force field [47,48]. The resulting model depicted that the region between the head domain and the Ig-like fold domain was mainly alpha-helical (Figure 3). In addition, we utilized the full-length lamin A/C generated by AlphaFold v2.0 and applied our color-coding scheme to the disease-associated residues [49] (Figure 3, bottom). The two models exhibit similar confidence scores (Supplementary Figure S1).

### 2.4. Generation of In Silico and In Vivo Models of Different Amino Acid Substitutions at the Same Position of LamC

Individuals with mutations in *LMNA* gene exhibit diverse disease symptoms Figure 1 [39,44]. The molecular genetic basis of this phenotypic diversity is not well understood. Possible non-mutually exclusive contributing factors include: (1) differences in chemical properties of the original amino acid and the one substituted and (2) difference in the genetic background of individuals.

To address this issue, we focused on the missense mutations in *LMNA* that cause different substitutions at position R249 of lamin A/C. These substitutions give clinically distinct types of muscular dystrophies that differ in age of onset, the subsets of muscles effected, and disease severity. Substitution of R for a Q at position 249 often results in AD-EDMD [50,51,52], as in the case studied here (Table 1). AD-EDMD is characterized by weakness and wasting of the distal muscles, joint contractures, especially of the ankles, elbows and neck that often presents after two years of age and sometimes into the second decade of life [16]. In addition, AD-EDMD is often accompanied by dilated cardiomyopathy with conduction defects [53]. By contrast, substitution of a W at position 249 often causes lamin-associated congenital muscular dystrophy (L-CMD) [54,55], as in the case studied here (Table 1). L-CMD is characterized by weakness and wasting of muscles prior to two years of age and can be accompanied by cardiac involvement [54].

To visualize potential effects of lamin A/C R249Q and R249W, we generated dimer models using AlphaFold v2.0 for wild-type and mutant lamins (Supplementary Figure S2). The wild-type dimer prediction showed two conformations: one extended and one possessing a bend after coil 1B. Both dimer conformations have similar confidence scores from AlphaFold v2.0 (Supplementary Figure S3). Similar extended and bent conformations were seen for dimers of R249Q and R249W with slight variations in the positions of the two monomers relative to one another in the region of the amino acid substitution (Supplementary Figure S2). In addition, the R249Q extended model showed an arch within the rod domain that was not predicted with R249W.

To directly compare the effects of these two different amino acid substitutions on lamin function, we turned to *Drosophila melanogaster*. The Drosophila genome encodes an orthologue of *LMNA* called *LaminC* (*LamC*). In addition, Drosophila provides a defined genetic background and relatively rapid genetic analyses. Our prior studies demonstrated that expression of mutant *LamC* transgenes, but not a wild-type, caused strikingly similar defects in fly muscles as those observed in humans [56,57]. Furthermore, the relative severity of the muscle defects caused by different *LamC* mutants corresponded to that observed in cases of *LMNA*-associated muscular dystrophy [22,57].

**Figure 4:**
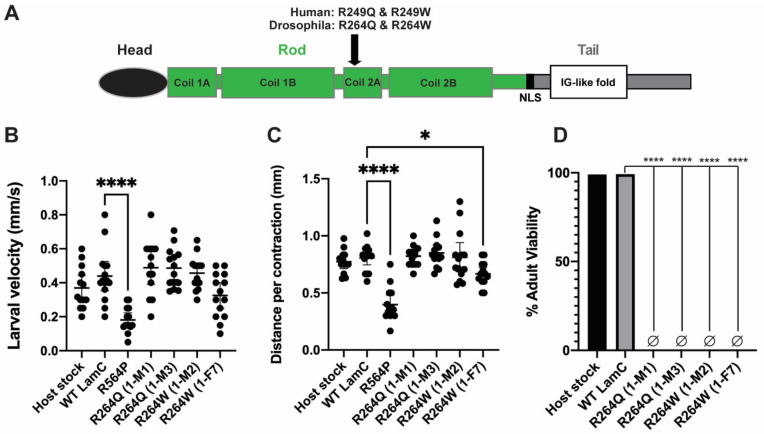
Effects of muscle-specific expression of mutant Drosophila *LamC* transgenes on larval motility and adult viability were analyzed. (**A**) Diagram of lamin A/C showing domains and location of the amino acid substitutions under study. (**B**) Velocity measurements were made on the host stock and transgenic larvae expressing either wild-type or mutant *LamC* exclusively in the larval body-wall muscles. For the transgenic larvae, the amino acid substitution is indicated along the X-axis; parentheses represent the strain designation). Velocity values are expressed as the mean ± standard deviation. *n* = 13–15 larvae per genotype. LamC R564P was previously shown to reduce motility and was used as a positive control. **** *p* < 0.0001 (**C**) Distance per contraction of third instar larvae were measured for the host stock and transgenic larvae expressing either wild-type *LamC* or mutant *LamC* exclusively in the larval body-wall muscles. Distance per contraction measurements are expressed as the means ± standard deviation. *n* = 13–15 larvae per genotype. * *p* < 0.05, **** *p* < 0.0001 (**D**) The percent adult viability was measured for the host stock and transgenic flies with larval body-wall muscle-specific expression of mutant *LamC*. Ø indicates zero adults were viable. *n* = 174–426 adults per genotype. **** *p* < 0.0001.

The amino acid R249 is conserved in Drosophila LamC and corresponds to R at position 264. Fly stocks were generated that express transgenes encoding wild-type LamC, R264Q and R264W (Figure 4A). The *LamC* transgenes were expressed using the Gal4/UAS system [58]. Briefly, flies expressing the yeast Gal4 transcription factor in muscle (Gal4 driver stock), were crossed to flies expressing a *LamC* transgene possessing an Upstream Activating Sequence (UAS) in the promoter region (responder stocks). In resulting progeny, the *LamC* transgenes were specifically expressed in muscle. For studies here, larval body wall muscle-specific expression was achieved using the *C57* Gal4 driver stock [59]. For indirect flight muscle-specific expression the *Act88F* Gal4 driver stock was used [60].

To ensure that defects observed were due to mutant *LamC* and not general over-expression, we quantified levels of LamC protein expressed in muscles. Total protein was extracted from muscles and used in western analyses. Two independent transformed lines were tested for each mutant. Western analysis revealed that the wild-type *LamC* transgenic flies possessed similar levels of total LamC as the host stock. No statistical difference was found between R264W (line 1-F7) and the transgenic stock expressing wild-type LamC (Supplementary Figure S4). Stock R264W (line 1-M2) showed a modest 1.78-fold increase compared to that expressing wild-type LamC (Supplementary Figure S4). Similarly, the transgenic stock expressing R264Q (line 1-M1) had nearly identical levels of LamC as that expressing wild-type. Transgenic stock R264Q (line 1-M3) showed a modest increase of 1.99-fold higher level than the wild-type control. Based on these results, transgenic stocks R264W (line 1-F7) and R246Q (line 1-1M) were used all subsequent analyses Thus, by selecting transgenic stocks that expressed nearly identical levels of LamC, defects were attributed to a specific amino acid substitution and not protein levels.

### 2.5. Larval Body Wall Muscle-Specific Expression of R264Q and R264W Did Not Affect Motility, but Caused Pupal Lethality

Drosophila body wall muscles have similar development and properties as human skeletal muscles. Their differentiation is determined by conserved transcriptional regulators [61,62,63]. The muscles form by the fusion of myoblasts, giving rise to multi-nucleated muscle fibers. The muscle fibers are arranged in a regular pattern and are connected to epidermal cells through attachment to tendon cells. Contraction of the larval body wall muscles is responsible for larval motility and aspects of morphogenesis during the early pupal stage [64].

Using the Gal4/UAS system, wild-type and mutant *LamC* transgenes were expressed in larval body wall muscles. Muscle function was assayed by placing third instar larvae in the center of an agarose filled petri dish and recording their motility by video. Analysis of the videos provided quantitative measurements of velocity and distance traveled per contraction. Larvae expressing wild-type LamC showed no difference in velocity and distance traveled per contraction compared to the host stock (Figure 4B,C). Surprisingly, larvae expressing either R264Q or R264W showed no significant differences in velocity and distance per contraction compared to that of wild-type larvae (Figure 4B,C). These results were unexpected since other Drosophila models of muscle laminopathies showed reduced motility [56]. Despite the lack of motility defects, expression of R264Q and R264W caused complete death at the pupal stage, whereas expression of wild-type LamC did not (Figure 4D). This suggests that the mutant *LamC* transgenes might cause altered gene expression during morphogenesis as has been observed with muscle-specific expression of a mutant lamin lacking the N-terminal head domain [65].

### 2.6. LamC R264Q and R264W Cause Nuclear Lobulation and Nuclear Envelope Protein Mislocalization

**Figure 5:**
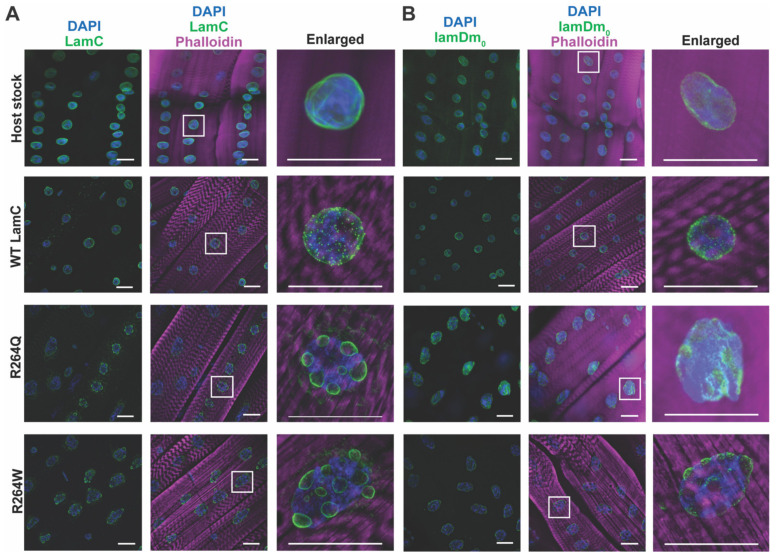
Expression of the *LamC R264Q* and *R264W* transgenes caused nuclear deformation. (**A**) Larval body-wall muscles expressing endogenous *LamC* (host stock), and either a wild-type *LamC* or mutant *LamC* transgene were fixed and stained with phalloidin (magenta), DAPI (blue) and antibodies to LamC (green). The scale bar represents 30 μm. (**B**) Larval body-wall muscles expressing either wild-type or mutant *LamC* were fixed and stained with phalloidin (magenta), DAPI (blue) and antibodies to lamDm_0_ (green). The scale bar represents 30 μm.

Given the lack of motility defects caused by R264Q/W, we examined LamC localization in the larval body wall muscles. The muscles were dissected, fixed, and stained with phalloidin (actin), DAPI (DNA), and antibodies to LamC (LC28.26; Developmental Studies Hybridoma Bank at the University of Iowa). Muscles expressing the wild-type *LamC* transgene showed LamC localization to the nuclear periphery as anticipated. By contrast, expression of the *R264Q* and *R264W* transgenes caused strikingly malformed myonuclei possessing numerous lobulations, with minor amounts of LamC observed in the perinuclear region (Figure 5A).

To determine if the nuclear lobulations altered the localization of other nuclear envelope proteins, larval body wall muscles were fixed and stained with phalloidin, DAPI and antibodies to lamin Dm_0_ (lamDm_0_), considered the Drosophila B-type lamin (ADL84.12; Developmental Studies Hybridoma Bank at the University of Iowa). Antibodies to lamDm_0_ showed nearly uniform staining at the nuclear periphery in muscles expressing the wild type *LamC* transgene. By contrast, lamDm_0_ staining was enriched in the lobulations of nuclei expressing mutant *LamC* (Figure 5B). Thus, muscle-specific expression of mutant versions of the A-type lamin altered myonuclear contour and caused mislocalization of the B-type lamin.

**Figure 6:**
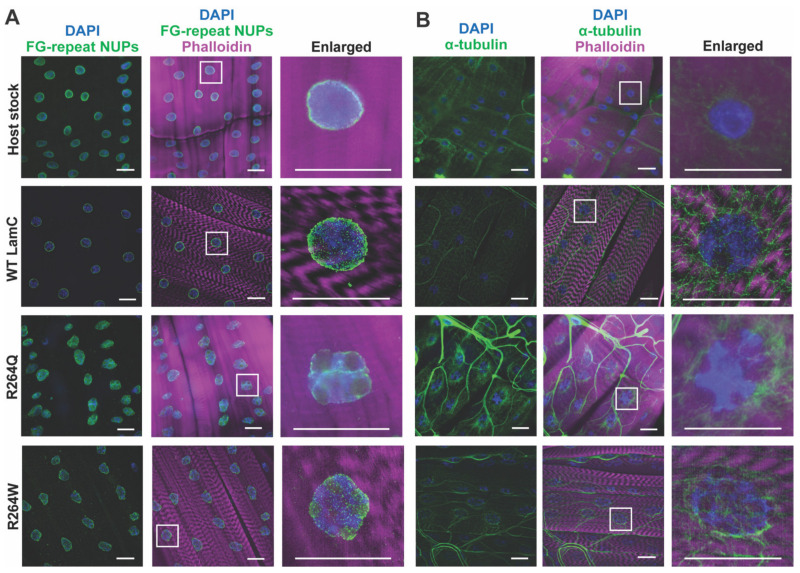
Nuclear deformation caused by expression of the *LamC R264Q* and *R264W* transgenes altered nuclear pore distribution, but not nuclear microtubule caging. (**A**) Larval body-wall muscles expressing endogenous *LamC* host stock and either a wild-type or mutant *LamC* transgene were fixed and stained with phalloidin (magenta), DAPI (blue) and antibodies to FG-repeat containing nuclear pore proteins (NUPs) (green). The scale bar represents 30 μm. (**B**) Larval body-wall muscles were fixed and stained with phalloidin (magenta), DAPI (blue) and antibodies to a-tubulin (green). The scale bar represents 30 μm. Lamins interact with nuclear pore proteins [26,27]. Therefore, we stained larval body wall muscles expressing wild-type and mutant LamC with antibodies that recognize FG-repeat containing nuclear pore proteins (NUPs) (Mab414; Covance). In muscles expression of wild-type *LamC*, the nuclear pore proteins were distributed evenly within the nuclear envelope (**A**). By contrast, muscles expressing the *R264Q* and *R264W* transgenes showed areas of concentration at the nuclear periphery.

In addition to nuclear envelope proteins, lamins influence cytoskeletal proteins through connections made through the Linker of Nucleoskeleton and Cytoskeleton (LINC) complex [26,28,29]. This connection is important for generating a cage of α-tubulin that surrounds and protects myonuclei from physical forces [66,67]. To determine if the nuclear lobulations altered the α-tubulin cage, we stained larval body wall muscles with antibodies to α-tubulin (AA4.3; Developmental Studies Hybridoma Bank at the University of Iowa). In wild-type muscles, α-tubulin surrounds the nuclear periphery, forming an obvious cage-like structure (Figure 6B). A similar pattern of staining is observed for myonuclei expressing R264Q and R264W. Thus, despite the striking nuclear envelope distortion caused by mutant LamC, the α-tubulin cage reminded intact.

Given that the lamin meshwork forms a scaffold for the attachment of genomic DNA packaged into chromatin, it is possible that substitutions at R264 disrupt such contacts [19,23,24,25]. To test for this, we analyzed the degree to which the fluorescent DAPI signal colocalized with the fluorescent signal from antibodies to LamC and lamDm_0_ using the JACoP plugin for Fiji [68,69]. Colocalization of DAPI with LamC was significantly reduced in the myonuclei of larvae expressing R264Q and R264W, relative to that of the control (Supplementary Figure S5A). Interestingly, colocalization of DAPI with lamDm_0_ was reduced in larvae expressing R264W, but not R264Q (Supplementary Figure S5B). These data imply that substitutions at R264 reduce contacts between the chromatin and lamina, which is predicted to alter gene expression.

### 2.7. Expression of R264Q and R264W in Indirect Flight Muscles Caused Wing Posturing Defects

**Figure 7:**
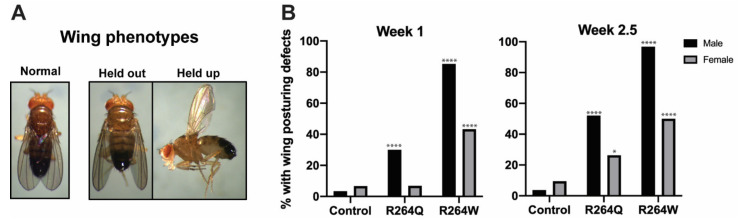
Expression of the *LamC R264Q* and *R264W* transgenes in the indirect flight muscles caused wing posturing defects. (**A**) Representative examples of the wing posturing defects. “Normal” indicates the typical wing posture of control flies expressing the *Act88F* Gal4-driver alone. “Held out” and “held up” are examples of wing posturing defects in adults expressing *LamC R264Q* in the indirect flight muscles. (**B**) Wing posturing defects were assayed in 1- and 2.5-week-old adults and plotted as a percentage of the total for each genotype. For 1-week-old adults *n* = 30–88; for 2.5-week-old adults *n* = 24–81. Transgenic flies expressing mutant lamins were compared to controls and separated by sex. * *p* < 0.05, **** *p* < 0.0001.

Since expression of the *R264Q* and *R264W* transgenes in larval body wall muscle caused no motility defects, we wondered what the effects would be on indirect flight muscle function. The indirect flight muscles are two sets of antagonistic muscles, the dorsal longitudinal and the dorsal ventral muscles, that control wing posturing and flight [70]. The transgenic stocks were crossed with the *Act88F* Gal4 driver, which expresses Gal4 exclusively in indirect flight muscles [62]. The resulting progeny were scored for abnormal wing posturing, a result of loss of indirect flight muscle function (Figure 7A) [71]. Almost none of the control flies showed abnormal wing posturing at 1 and 2.5 weeks of age. By contrast, 98.6% and 52.1% of 2.5-week-old males expressing the *R264W* and *R264Q* transgenes, respectively, showed abnormal wing posturing (Figure 7B, Supplementary Table S3). Adults with wing posturing defects displayed both a held-out and held-up wing phenotype, both indicative of loss of indirect flight muscle function (Figure 7A). Interestingly, the effect was more pronounced in males. At both 1 and 2.5 weeks of age, males and females expressing the *R264W* transgene showed greater numbers of wing posturing defects than flies expressing the *R264Q* transgene, suggesting that severity is linked to the nature of the amino acid substitution.

## 3. Discussion

We performed an extensive genotype-phenotype analysis using 315 disease-associated amino acid substitutions that were obtained from two publicly available databases (http://www.umd.be/LMNA/ accessed 1 June 2020; https://databases.lovd.nl/shared/varants/LMNA/unique accessed 1 June 2020) (Figure 1). We color-coded amino acids altered in laminopathies and mapped them onto the three existing three-dimensional lamin A/C structures (PDB: 6SNZ; 1X8Y; 1IVT). There was no apparent connection between the disease phenotypes and the position of an altered amino acid. Our findings are consistent with those noted using smaller datasets [72,73].

A prior study found that substitutions of amino acids within the interior of the Ig-like fold were more likely to cause muscular dystrophy, possibly due to distortion of the barrel-like structure of this domain [41]. These authors also found that substitution of amino acids on the exterior or surface of the Ig-like fold were more likely to be associated with lipodystrophy, possibly due to the disruption of partner protein interactions [41]. We expanded this analysis by using a greater number of laminopathy cases now available in databases. Based on our analyses, the predicted solvent accessibility of the affected amino acid did not correlate with muscular dystrophy or lipodystrophy (Supplementary Table S1).

We placed our color-coded portions of lamin A/C on a newly generated full-length model of lamin A (Figure 3). Our model was generated by splicing known structures for portions of lamin A into a structure predicted by trRosetta [45] (Figure 3, top). Recently, a full-length lamin A structure was predicted by AlphaFold v2.0 [49]. There is general agreement between our model and that generated by AlphaFold v2.0 (Figure 3, bottom). Both models predicted that the head domain (residues 1–33) is partially disordered with some alpha helical character. Both models agree that Coil 1A, Linker 1, and Coil 1B are alpha helical. There is some uncertainty on the conformation for Linker 2 (residues 219–242) with trRosetta showing a linear confirmation, whereas AlphaFold v2.0 favored a bend. Both models determined Coil 2 (residues 243–383) to be alpha helical, which is where R249 maps. In addition, both models agree that residues 428–545 generate an Ig-like fold domain. The most significant difference between the two models is within the tail region (residues 384–427 and 546–664) that flanks the Ig-like fold. The trRosetta algorithm predicted the flanking regions can form a globular domain whereas AlphaFold v2.0 predicted these regions to be disordered. The differences between these two models in these flanking regions suggest a lack of stability. Perhaps the conformation of these regions is dependent on protein interactions and/or the cellular environment. Experimental determination of a full-length lamin A/C could resolve these differences; however, conformational heterogeneity is likely to present challenges.

The wild-type lamin A/C dimer structure predictions compare well with the experimentally derived structure of a lamin A/C dimer [74]. The AlphaFold v2.0-predicted extended dimer structure shows concordance with the experimental model [74]. The AlphaFold v2.0 bent structure shows a region of disorder that corresponds to a disordered region in the experimental model, consistent with conformational flexibility. However, the experimental model of the dimer showed association between the Ig-like folds, which was not present in any of the top five AlphaFold v2.0 dimers. Since AlphaFold v2.0 makes use of coevolutionary data (from multiple sequence alignments), the lack of association in the Ig-like folds in the predicted lamin A/C dimer reflects a lack of evidence for coevolution of an Ig-like fold dimer interface.

The R249Q amino acid substitution perturbed packing of the extended dimer prediction in the region of the substitution, causing an arched conformation (Supplementary Figure S2). In contrast, the R249W amino acid substitution produced an extended dimer structure similar to that of the wild-type. The R249Q and R249W bent structure predictions did not show any discernable disruptions in the association of the dimer compared to that of the wild-type bent structure. It is important to note the ability of AlphaFold v2.0’s to model single amino acid substitutions has not been validated. Therefore, the structure predictions for R249Q and R249W dimers are starting points for future investigations (e.g., using molecular dynamics simulations).

The Ig-like fold domain of lamin A/C has been well-studied and many interaction partners have been experimentally determined [26,44]. Using in silico methodology, we identified nine potential binding pockets [43] (Figure 2). Interestingly, seven of the predicted binding pockets mapped to positions of disease-causing amino acids. For example, substitution at position 520, which overlaps two predicted binding pockets (Figure 2), caused loss of binding with DDX43 (DEAD-Box Helicase 43), an ATP-dependent RNA helicase (Supplementary Table S2). The loss of partner protein interactions might contribute to disease pathology.

Of the 205 amino acid positions investigated here, 42 are sites where substitution with different amino acids causes clinically distinct diseases. It is unclear if the nature of the replacement amino acid or differences in an individual’s genetic background cause disease variation in these cases. Here, we focused on R249W, which is the most prevalent amino acid substitution in cases of L-CMD [72,75], and R249Q, which is more commonly observed with AD-EDMD [50,51]. The substitution of the charged R residue with the aromatic side chain of W or the uncharged polar residue Q might alter lamin homo-dimerization.

To determine if the different chemical properties contribute to muscle disease severity, we compared the effects of R249Q and R249W modeled in Drosophila LamC. Using Drosophila allowed us to minimize genetic background effects and test the functions of two mutant lamins that differ by only one amino acid. Both mutant lamins caused defects when expressed in larval body wall muscles of an otherwise wild-type organism, strongly suggesting that they function as dominant negatives. Both mutant lamins altered nuclear envelope morphology by producing nuclear lobulations (Figure 5). Given that the amino acid substitutions are in the rod domain, it is possible that the lobulations arise from alterations in lamin homo-dimerization, resulting in irregularities in the lamina meshwork, as our modeling would suggest (Supplementary Figure S5) [13]. The lobulations are distinctly different from what we have observed upon expression of single amino acid substitutions within the LamC Ig-like fold domain. In these cases, the nuclei retained a mostly round shape [56,57]. It is worthwhile to note that human iPSC-derived myotubes expressing R249W showed lamin nuclear aggregation and misshapen nuclei [76]. Despite the fact that the lobulated nuclei caused mislocalization of the B-type lamin and nuclear pores in larval body wall muscles (Figure 5 and Figure 6), motility was surprisingly not affected (Figure 4B,C). In our prior work, single amino acid substitutions in the Ig-like fold domain reduced larval motility [57]. It is unclear what the molecular basis is for the differing effects on larval motility. However, mutant lamins that altered larval motility exhibited cytoplasmic aggregation of LamC [57]. Taken together, these finding suggest that different mutant lamins have different pathomechanisms. Supporting this idea, in addition to functions at the nuclear envelope, lamins have distinct roles in the nucleoplasm [28,77]. Therefore, the amino acid substitutions studied here might impact non-nuclear envelope functions.

The Drosophila models expressing the equivalent of R249W and R249Q, which are R264W and R264Q, respectively, exhibited premature death at the pupal stage by mechanisms that are unclear (Figure 4D). One possibility is that despite normal motility, the larval body wall muscles do not contract properly during the pupal stage to carry out morphogenesis [64]. Another possibility is that the lobulated nuclei cause disorganization of the genome, resulting in altered gene expression. This idea is supported by the reduction of colocalization between DNA and lamins (Supplementary Figure S5). Previously, we found that expression of a headless (ΔN) LamC in Drosophila larval body wall muscles caused reduced expression of steroid hormone regulated genes required for morphogenesis during the pupal stage [65]. These possibilities are non-mutually exclusive whereby both physiological functioning and gene expression could be simultaneously affected by mutant lamins.

In contrast to our results obtained in larval body wall muscles, expression in indirect flight muscles highlighted differences between R264Q and R264W. Expression of both mutant lamins produced progressive wing posturing defects that are indicative of loss of muscle function. At 1 and 2.5 week of age, a greater number of adults expressing R264W showed wing posturing defects compared to those expressing R264Q (Figure 7B). This held true for both males and females. In both genotypes, males were more affected than females, for reasons that are unclear (Figure 7B). Thus, our finding suggest that R249W is more detrimental to muscle function than R249Q. Consistent with R249W having severe effects, a recent clinical study found R249W to have a worse prognosis compared with four other non-truncating *LMNA* mutations [72].

## 4. Materials and Methods

### 4.1. In Silico Analyses

#### 4.1.1. Mapping Disease Related Amino Acid onto Lamin A/C

Three dimension-structural information was available for three portions of human lamin A/C in the RCSB Protein Data Base (6SNZ; 1X8Y; 1IVT) [78,79,80]. The PBD files were loaded into PyMol for in silico analysis (The PyMOL Molecular Graphics System, Version 2.0 Schrodinger, LLC, New York, NY, USA). The amino acids associated with disease were color coded according to a particular disease or multiple diseases (Figure 1).

#### 4.1.2. Deep Learning Prediction of Full-Length Lamin

To generate a full-length lamin A, experimentally solved domains of the overall structure were linked together using a full-length structure from a deep learning algorithm. Structural models obtained from the RCSB Protein Data Bank were locally optimized using the Amber ‘99 forcefield to allow backbone relaxation and favorable torsions [46]. Initial optimization was performed with a root mean square (RMS) convergence criterion of 0.8 kcal/mol/Å. Many-body energy expansions were applied to the refined structures using the AMOEBA forcefield to optimize side-chain placement and improve MolProbity scores [81]. The optimized structures were locally optimized again with an RMS convergence criterion of 0.1 kcal/mol/Å to finalize backbone torsions with the optimized sidechain placement.

The full-length structure was predicted from the amino acid sequences using trRosetta without templates [45]. The model with the highest TM-score (0.377) was chosen. The full-length predicted structure was aligned with the optimized strcutural models using PyMol, and the overlapping residues were removed from the full-length structure. The final structure, including experimental models spliced in, was locally optimized using polarizable AMOEBA forcefield [51,52]. Dimers of lamin A/C and its variants were generated using ColabFold, a software package that simplifies use of AlphaFold v2.0. The top five structures with the highest pLDDT scores were generated using 15 recycles.

#### 4.1.3. Determination of Potential Partner Protein Binding Pockets within the Ig-Like Fold Domain

To determine potential partner protein interaction pockets, amino acid sequences of the human lamin A/C Ig-like fold domain (428–529) were submitted to the online software POCASA version 1.1 [43]. Default settings were used, with the exception of probe radius, which was changed from 2 Å to 4 Å. The output PDB file contained predicted binding pockets. The position of these pockets was overlain on a surface model of Ig-like fold domain generated using PyMol in which amino acid residues associated with disease were indicated by colors (Figure 2).

#### 4.1.4. Determination of Surface and Buried Resides within the Ig-Like Fold Domain

Using in silico methods, amino acid residues in the Ig-like fold associated with muscular dystrophy or lipodystrophy were determined to be on the surface or buried within the folded structure. The PDB file for the Ig-like fold domain (1IVT) was submitted to GETAREA 1.0, a program that determines relative Solvent Accessible Surface Area (SASA) scores [46]. The side chains of the muscular dystrophy and lipodystrophy-associated residues were analyzed, and those with a relative SASA % ≤ 20% were designated “exposed”. Side chains with a relative SASA % > 20% were designated “exposed”.

### 4.2. In Vivo Analyses

#### 4.2.1. Drosophila Culturing

Drosophila stocks were raised at 25 °C on standard sucrose/cornmeal medium [82]. A full-length *LamC* cDNA was used in site directed mutagenesis (QuickChange, Agilent, Santa Clara, CA, USA) to generate base changes that were predicted to encode R264Q and R264W. The resulting DNA was closed into the pUAST Drosophila transformation vector [58]. Transgenic stocks were generated using standard P-element transformation (BestGene Inc., Chino Hills, CA, USA). Transgenic stocks were made homozygous prior to using in crosses with Gal4 driver stocks.

#### 4.2.2. Western Analysis

Protein was extracted from three independent sets of larvae for each genotype tested. For each sample, two muscle filets were hand-dissected from third instar larvae, then ground in 2X Laemmli grinding buffer (125 mM Tris HCL, 20% glycerol, 4% SDS, 0.005% bromophenol blue, final pH 6.8) plus 20 mM DTT. The samples were then boiled for five minutes, followed by 15,000 rpm centrifugation for five minutes. The resulting supernatants were taken as protein lysates, which were separated on NuPage 4–12% BisTris gradient gels using 1X MES/SDS. The gels were transferred to nitrocellulose membranes and blocked 5% BSA/TBS. The membranes were incubated with LI-COR Revert 700 Total Protein Stain according to protocol. Drosophila LamC was detected using LC28.26 (University of Iowa Developmental Biology Hybridoma Bank, University of Iowa) (1:2000 dilution in 1% BSA/TBS-T) overnight at 4 °C. Following several TBS-T washes, the membranes were incubated with the secondary antibody DyLight 800 (LI-COR) (goat anti-mouse, 1:10,000 dilution in 1% BSA/TBS-T) for 1 h at room temperature. Membranes were imaged using a LI-COR Odyssey CLx scanner and quantified using Image Studio. LamC levels were normalized to total protein levels, then made relative to the level of LamC produced in the stock possessing a wild-type *LamC* transgene.

#### 4.2.3. Larval Motility Assays

Third instar larvae in groups of 4–6 were placed on a 15 cm petri dish containing solidified 0.1% agarose plates at room temperature for two minutes of equilibration. Larvae were then transferred to a second 15 cm petri dish containing solidified 0.1% agarose and two-minute video recording were made using a cell phone. Larval velocity was calculated by examining the distance the larvae traveled in one ten second period. Only larvae traveling in a straight line were analyzed. Larval distance per contraction was calculated by dividing the distance traveled by the total number of contractions in the same ten second interval. Stationary larvae were not included in the calculations.

#### 4.2.4. Larval Body Wall Muscle Cytology

Larval body wall muscles were prepared and stained according to published procedures [83]. After fixation, the muscle filets were blocked in 1X PBS overnight at 4 °C. The muscles were stained with Texas Red™-X phalloidin (1:400 dilution; Invitrogen, Waltham, MA, USA) in permeabilization buffer (1X PBS; 0.5% TX100, 5 mM MgCl_2_) containing 0.5% boiled fish skin gelatin (FSG). Drosophila LamC was detected using LC28.26 (anti-mouse, 1:200 dilution; Developmental Studies Hybridoma Bank at the University of Iowa), Drosophila lamDm_0_ was detected using ADL84.12 (anti-mouse, 1:400 dilution; Developmental Studies Hybridoma Bank at the University of Iowa), Drosophila peripheral nuclear pore proteins were detected using mouse monoclonal antibody Mab414 (1:2000 dilution; Covance, Princeton, NJ, USA). Drosophila α-tubulin was detected using mouse monoclonal antibody AA4.3 (1:200 dilution; Developmental Studies Hybridoma Bank at the University of Iowa). All primary antibodies were diluted in permeabilization buffer containing 0.5% boiled FSG and incubated overnight at 4 °C. Primary antibodies were recognized with secondary antibody Alexa-Fluor 488 (donkey anti-mouse, 1:400 dilution; Invitrogen) in permeabilization buffer containing 0.5% boiled FSG for 2 h at room temperature. Imaging was performed using a Leica DMLB fluorescent microscope with a 40X oil objective or a Leica Thunder microscope with a 60X oil objective.

#### 4.2.5. Colocalization of Fluorescent Signals

Two-channel fluorescent microscope images of larval body wall muscle were analyzed using the JACoP plugin for Fiji [68,69]. The images were split into separate channels, one containing the signal for DAPI and the other containing the signal for either LamC or lamDm_0_. Background was previously subtracted from each channel. Using the LamC/lamDm_0_ channel, regions of interest (ROIs) were defined by enclosing individual nuclei with the freehand tool. Care was taken to exclude as much cytoplasmic lamin as possible, as there could be no colocalization of the cytoplasmic lamin with the DAPI. The ROIs were added to the ROI manager. Each ROI was cropped out of the two channels, resulting in an image containing DAPI representing the nucleus and an image containing lamin. These cropped images were analyzed using the JACoP plugin [68]. Threshold values were calculated automatically by the software. The Pearson’s correlation coefficient between the lamin and DAPI was recorded for each nucleus. Ten nuclei were analyzed per genotype.

#### 4.2.6. Adult Viability Assays

Transgenic flies possessing wild-type and mutant *LamC* transgenes were crossed to flies from the *C57* Gal4 driver [59]. After four days, the adults were removed from the vials. After two weeks live adults and dead pupae were counted for all genotypes. Adult percent viability was calculated as $\frac{\#\mathrm{living}\;\mathrm{adults}}{\#\mathrm{living}\;\mathrm{adults}+\#\mathrm{dead}\;\mathrm{pupae}}\times 100$.

#### 4.2.7. Analysis of Indirect Flight Muscle Function

Transgenic flies possessing wild-type and mutant *LamC* transgenes were crossed to flies from the *Act88F* Gal4 driver stock (Bloomington Stock Center, Bloomington, IN, USA). After four days, the adults were removed from the vials. Newly emerged adults were collected and aged 1 and 2.5 weeks prior to examination. Males and females were raised separate vials. No statistical difference in the wing posturing between the two conditions was noted. We found that carbon dioxide, used to put flies asleep under the microscope, altered their wing posturing. Therefore, we used carbon dioxide to collect 5–6 adults per empty vial and allowed them to fully recover before scoring for wing posture.

### 4.3. Statistical Analyses

Graphpad Prism (v9.2.0, Graphpad Software, La Jolla, CA, USA) was used to calculate and plot the mean and standard deviation for larval velocity, larval distance per contraction, and Western analyses. Graphpad Prism was also used for plotting adult viability and indirect flight muscle disease. Statistical significances for larval velocity, larval distance per contraction, Western analyses, and colocalization studies were determined using an ANOVA followed by a Dunnett correction. Statistical significances for solvent accessibility of the Ig-like fold, adult viability, and indirect flight muscle disease were determined using the Fisher’s exact test. Differences between conditions were considered significant at levels of *p* < 0.05, *p* < 0.01, *p* < 0.001, and *p* < 0.0001. Any non-significant differences between conditions were left unmarked. Graphs for figures were generated with Graphpad Prism and tables were made using Word (Microsoft).

## Figures and Tables

**Table 1:** Clinical data of individuals with muscular dystrophy that served as a basis for this study.

Patient #	DNA Mutation	Gene Affected	Amino Acid Substitution	Age of Onset	Symptoms (Age of Symptoms)	CK Levels (Age Taken)	Clinical Diagnosis (Age of Diagnosis)
1	c.746G>A	*LMNA*	R249Q	2 years	Lordosis, toe walking, abnormal gait, lack of reflex, lower extremity weakness (2 yo) Low resting heart rate (24 yo) Atrial fibrillation (27 yo)	582 U/L (27 yo)	Polymyositis (2 yo) AD-EDMD (27 yo)
2	c.745C>T	*LMNA*	R249W	5 months	Hyperlordosis, dropped head, poor feeding (5 mo)	1697 U/L (5 mo)	L-CMD (5 mo)

## Data Availability

The data presented in this study are available on request from the corresponding author. Databases referenced in this study may be accessed at http://www.umd.be/LMNA/ accessed on 1 June 2020; https://databases.lovd.nl/shared/varants/LMNA/unique accessed on 1 June 2020.

## Supplementary Materials

The following are available online at https://www.mdpi.com/article/10.3390/ijms222011226/s1, Supplementary Figure S1: Lamin A/C monomer models were generated using AlphaFoldv2.0 through ColabFold, Supplementary Figure S2: The position of R249 was mapped onto the lamin A/C dimers, Supplementary Figure S3: Lamin A/C dimer models were generated using Al-phaFoldv2.0 through ColabFold, Supplementary Figure S4: LamC protein levels were quantified by Western, Supplementary Figure S5: Colocalization of DAPI with LamC and lamDm0 was an-alyzed, Supplementary Table S1: Amino acid residues associated with muscular dystrophy and lipodystrophy were analyzed for side chain positions within the Ig-like fold, Supplementary Table S2: Predicted protein interaction pockets in the lamin A/C Ig-like fold domain possess amino acids that are necessary for partner protein interactions, Supplementary Table S3: Abnormal wing pos-turing caused by R264Q/W was analyzed.

## Abbreviations

AD-EDMD	Autosomal dominant Emery–Dreifuss muscular dystrophy
AMOEBA	Atomic Multipoles Optimized Energetics for Biomolecular Applications
BSA	Bovine serum albumin
DDX43	Dead-box helicase 43
DTT	Dithiothreitol
FSG	Fish skin gelatin
IFM	Indirect flight muscle
Ig	Immunoglobulin
L-CMD	*LMNA*-related congenital muscular dystrophy
LAD	Lamin-associated domain
LamC	Lamin C
lamD_m_0	lamin D_m_0
LINC	Linker of nucleoskeleton and cytoskeleton
NUP	Nuclear pore protein
PBS	Phosphate buffer solution
POCASA	POcket-CAvity Search Application
R249Q	LMNA p.Arg249Gln
R249W	LMNA p.Arg249Trp
R264Q	Lamin C p.Arg264Gln
R264W	Lamin C p.Arg264Trp
RMS	Root mean square
ROI	Region of interest
SASA	Surface-accessible solvent area
SDS	Sodium dodecylsulfate
TBS	Tris-buffered saline
TBS-T	Tris-buffered saline-tween
TM	Template modeling
UAS	Upstream activation sequence
WT	Wild-type
