# Supplementary material for: In Silico and In Vivo Analysis of Amino Acid Substitutions That Cause Laminopathies
Source: Int J Mol Sci. 2021 Oct 18;22(20):11226. doi: 10.3390/ijms222011226 (PMC8536974; doi:10.3390/ijms222011226)
Supplement: Supplementary file 1 [file ijms-22-11226-s001.zip › Supplemental Figure Legends.pdf]

Supplementary Figure S1. Lamin A/C monomer models were generated using AlphaFoldv2.0 through ColabFold. (A) The monomer was generated using the AlphaFoldv2.0 extended model. (B) The monomer was generated using the AlphaFoldv2.0 bend model. The color of the residues indicates the confidence level of the predicted structure.

Supplementary Figure S2. The position of R249 was mapped onto the lamin A/C dimers. (A). Lamin A/C dimer models possessing the R249Q and R249W amino acid substitutions were generated using the AlphaFoldv2.0 extended model of the lamin A/C monomer. A magnified view of the region surrounding the amino acid substitution is shown at the right. (B) Lamin A/C dimer models were possessing the R249Q and R249W amino acid substitutions were generated using the AlphaFoldv2.0 bent model of the lamin A/C monomer. A magnified view of the region surrounding the amino acid substitution is shown at the right.

Supplementary Figure S3. Lamin A/C dimer models were generated using AlphaFoldv2.0 through ColabFold. (A) The dimer was generated using the AlphaFoldv2.0 extended model. (B) The dimer was generated using the AlphaFoldv2.0 bend model. The color of the residues indicates the confidence level of the predicted structure.

Supplementary Figure S4. Western analysis of LamC levels in the transgenic stocks. (**A**) Proteins were isolated from larval body-wall muscle and separated by size on an SDS-PAGE gel, transferred to nitrocellulose and stained with REVERT (LI-COR) total protein stain for normalization among the samples (right image). The same membrane was stained with antibodies to LamC and DyLight 800 (ThermoScientific) for detection. LamC is indicated by arrows. A third LamC protein species that was only present in muscle expressing mutant LamC is indicated by an asterisk and included in the quantification. The bracket indicates possible non-specific binding of the antibody to proteins in all samples, including the control. Alternatively, these bands might represent common lamin breakdown products that occur in all genetic backgrounds. (**B**) Relative levels of LamC were plotted. Data from relative intensity of the LamC bands was measured and normalized for differences in total protein among the samples and then to the levels present in the wild-type *LamC* transgenic flies. $n = 3$ independent biological samples.

Supplementary Figure S5. Colocalization of DAPI with LamC and lamDm$_0$ was analyzed. (**A**) Larval body wall muscle nuclei of transgenic larvae expressing either wild-type *LamC* or mutant *LamC* were analyzed for the colocalization of DAPI with LamC using the JACoP plugin for Fiji. The amino acid substitution expressed in the larvae is indicated along the X-axis. Pearson's correlation coefficient values obtained by comparing the DAPI signal with the lamin signal are expressed as the mean ± standard deviation. $n = 10$ nuclei per genotype. ** $p < 0.01$, **** $p < .0001$ (**B**) Larval body wall muscle nuclei of transgenic larvae expressing either wild-type *LamC* or mutant *LamC* were analyzed for the colocalization of DAPI with lamDm$_0$ using the JACoP plugin for Fiji. The amino acid substitution expressed in the larvae is indicated along the X-axis. Pearson's correlation coefficient values between the DAPI signal and lamDm$_0$ signal are expressed as the mean ± standard deviation. $n = 10$ nuclei per genotype. *** $p < 0.001$.
