# Supplementary material for: In Silico and In Vivo Analysis of Amino Acid Substitutions That Cause Laminopathies
Source: Int J Mol Sci. 2021 Oct 18;22(20):11226. doi: 10.3390/ijms222011226 (PMC8536974; doi:10.3390/ijms222011226)
Supplement: Supplementary file 1 [file ijms-22-11226-s001.zip › Table S1.pdf]

Supplementary Table S1. Analysis of surface exposed and buried side chains of Ig-like fold amino acid residues associated with muscular dystrophy and lipodystrophy.

	Side chain exposed	Side chain buried
Muscular dystrophy	18	17
Lipodystrophy	4	8

Correlation of side chain exposure and specific disease: p = 0.3314
