# Supplementary material for: In Silico and In Vivo Analysis of Amino Acid Substitutions That Cause Laminopathies
Source: Int J Mol Sci. 2021 Oct 18;22(20):11226. doi: 10.3390/ijms222011226 (PMC8536974; doi:10.3390/ijms222011226)
Supplement: Supplementary file 1 [file ijms-22-11226-s001.zip › Table S2.pdf]

Supplementary Table S2. Predicted protein interaction pockets in the lamin A/C Ig-like fold domain possess amino acids that are necessary for partner protein interactions.

Pocket Rank	Overlapping disease-related amino acid residue(s)	Lost binding partners	Associated disease(s)
1	481	CCNG1, MORF4L1, ZNF3	LGMD-AD, cardiac conduction block
	512	ZNF3	LGMD, EDMD
2	520	ABCA9, ANXA6, C4orf6, CENPP, CETN3, DDX43, DERL1, MORF4L1, MYADM, PARPBP, PKD2, RANBP9, RGS18, SPANXC, SPANXD, STAC2, TM4SF20, UBE2Q1, ZNF138, ZNF3	EDMD, CMD, LGMD, DCM
3			
4	523	ANXA6, MORF4L1, SEC22A, ZNF3	LGMD, DCM
5	472		LGMD
	527	CENPP, MORF4L1, SEC22A, TMEM74, ZNF3, ZNF569	HGPS, MAD, progeria, EDMD, LGMD
	529	CCNG1, MORF4L1, TOR1AIP1, ZNF3	MAD
6	467		Werner syndrome
7	520	ABCA9, ANXA6, C4orf6, CENPP, CETN3, DDX43, DERL1, FCER1A, GIN1, MORF4L1, MYADM, PARPBP, PKD2, RANBP9, RGS18, SPANXC, SPANXD, STAC2, TM4SF20, UBE2Q1, ZNF138, ZNF3	EDMD, CMD, LGMD, DCM
8			
9	488	MORF4L1, ZNF3	Isolated atrial fibrillation
	493		EDMD
