# Supplementary material for: In Silico and In Vivo Analysis of Amino Acid Substitutions That Cause Laminopathies
Source: Int J Mol Sci. 2021 Oct 18;22(20):11226. doi: 10.3390/ijms222011226 (PMC8536974; doi:10.3390/ijms222011226)
Supplement: Supplementary file 1 [file ijms-22-11226-s001.zip › Table S3.pdf]

Supplementary Table S3. Analysis of abnormal wing posturing caused by R264Q/W.

Transgenic stock (line)	n	% wing defect	Transgenic stock (line)	n	% wing defect	*p*-value	Significance
Week 1 Comparisons - Male							
Control	88	3.41	R264W (1-F7)	34	85.3	<0.0001	****
Control	88	3.41	R264Q (1-M1)	50	30	<0.0001	****
R264W (1-F7)	34	85.3	R264Q (1-M1)	50	30	<0.0001	****
Week 1 Comparisons - Female							
Control	75	6.67	R264W (1-F7)	30	43.3	<0.0001	****
Control	75	6.67	R264Q (1-M1)	58	6.90	>0.9999	ns
R264W (1-F7)	30	43.3	R264Q (1-M1)	58	6.90	<0.0001	****
Week 2.5 Comparisons - Male							
Control	81	3.7	R264W (1-F7)	31	96.8	<0.0001	****
Control	81	3.7	R264Q (1-M1)	48	52.1	<0.0001	****
R264W (1-F7)	31	96.8	R264Q (1-M1)	48	52.1	<0.0001	****
Week 2.5 Comparisons - Female							
Control	74	9.46	R264W (1-F7)	24	50	<0.0001	****
Control	74	9.46	R264Q (1-M1)	57	26.3	0.0171	*
R264W (1-F7)	24	50	R264Q (1-M1)	57	26.3	0.0691	ns
Week 1 vs 2.5 Comparisons – Male							
Control	88	3.41	Control	81	3.7	>0.9999	ns
R264W (1-F7)	34	85.3	R264W (1-F7)	31	96.8	0.1996	ns
R264Q (1-M1)	50	30	R264Q (1-M1)	48	52.1	0.0393	**
Week 1 vs 2.5 Comparisons – Female							
Control	75	6.67	Control	74	9.46	0.5633	ns
R264W (1-F7)	58	43.3	R264W (1-F7)	57	50	0.7843	ns
R264Q (1-M1)	30	6.90	R264Q (1-M1)	24	26.3	0.0058	**

* $p < 0.05$, ** $p < 0.01$, **** $p < 0.0001$
