# Supplementary figures and images for: In Silico and In Vivo Analysis of Amino Acid Substitutions That Cause Laminopathies
Source: Int J Mol Sci. 2021 Oct 18;22(20):11226. doi: 10.3390/ijms222011226 (PMC8536974; doi:10.3390/ijms222011226)

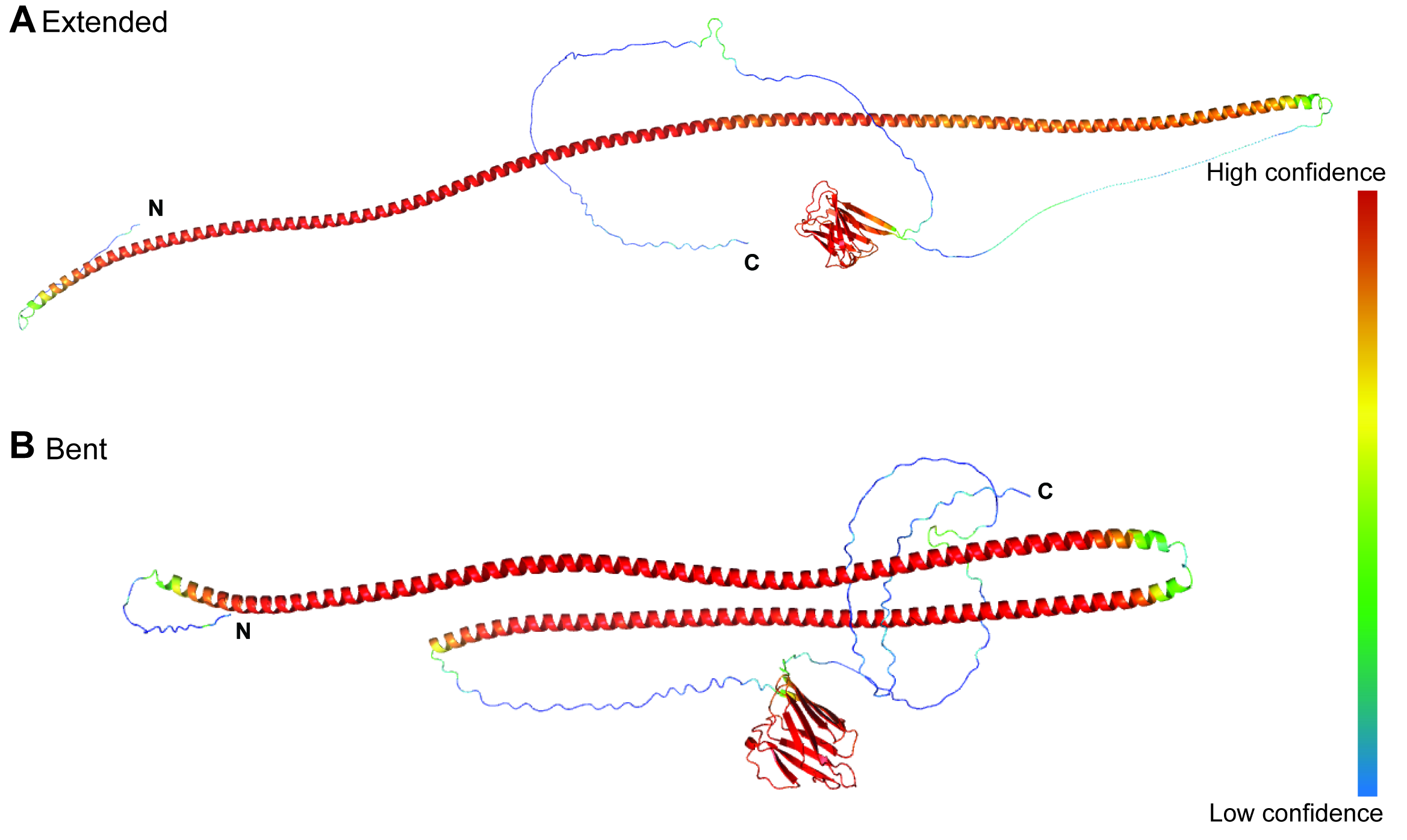

Supplement: Supplementary file 1 [file ijms-22-11226-s001.zip › Figure S1.tif]

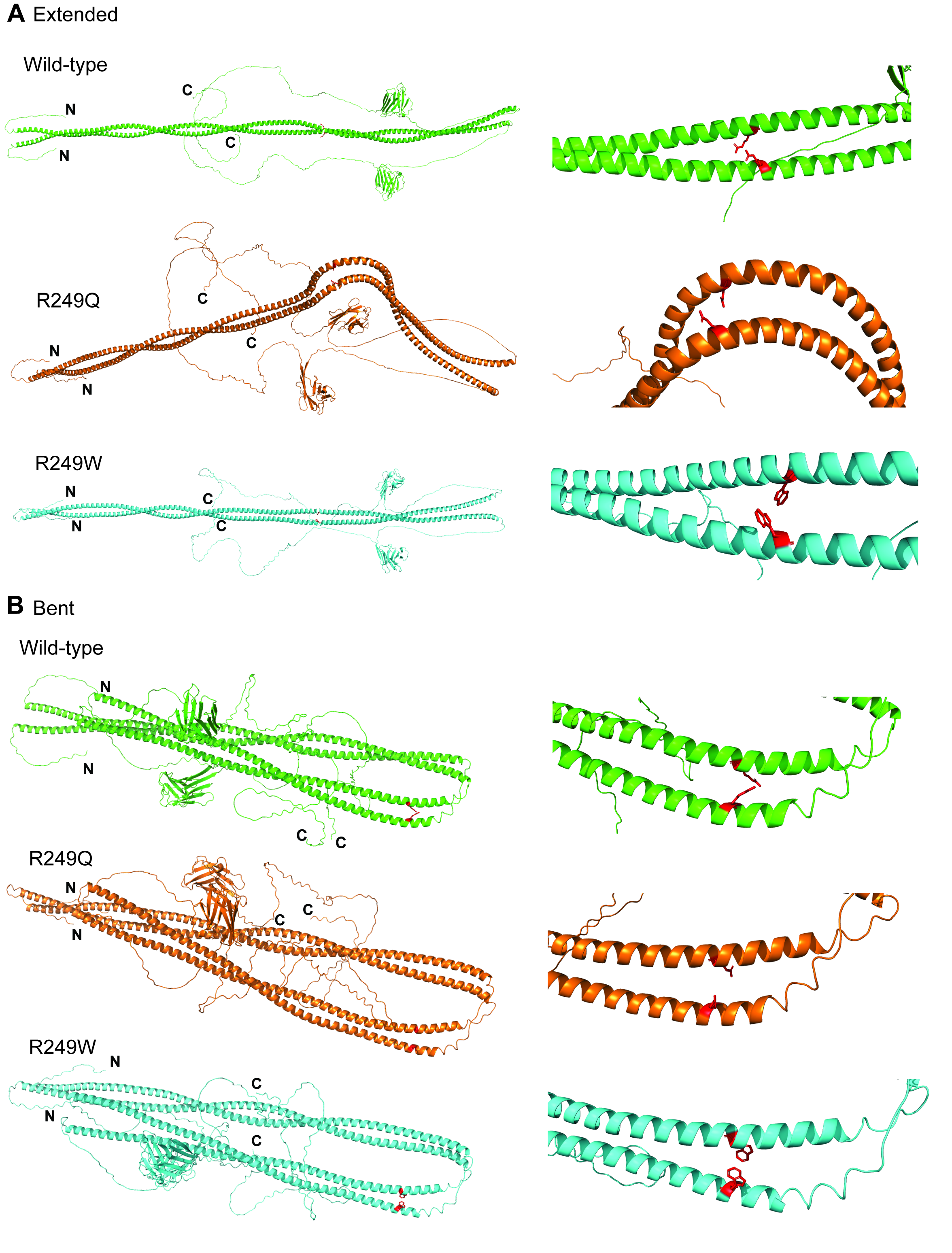

Supplement: Supplementary file 1 [file ijms-22-11226-s001.zip › Figure S2.tif]

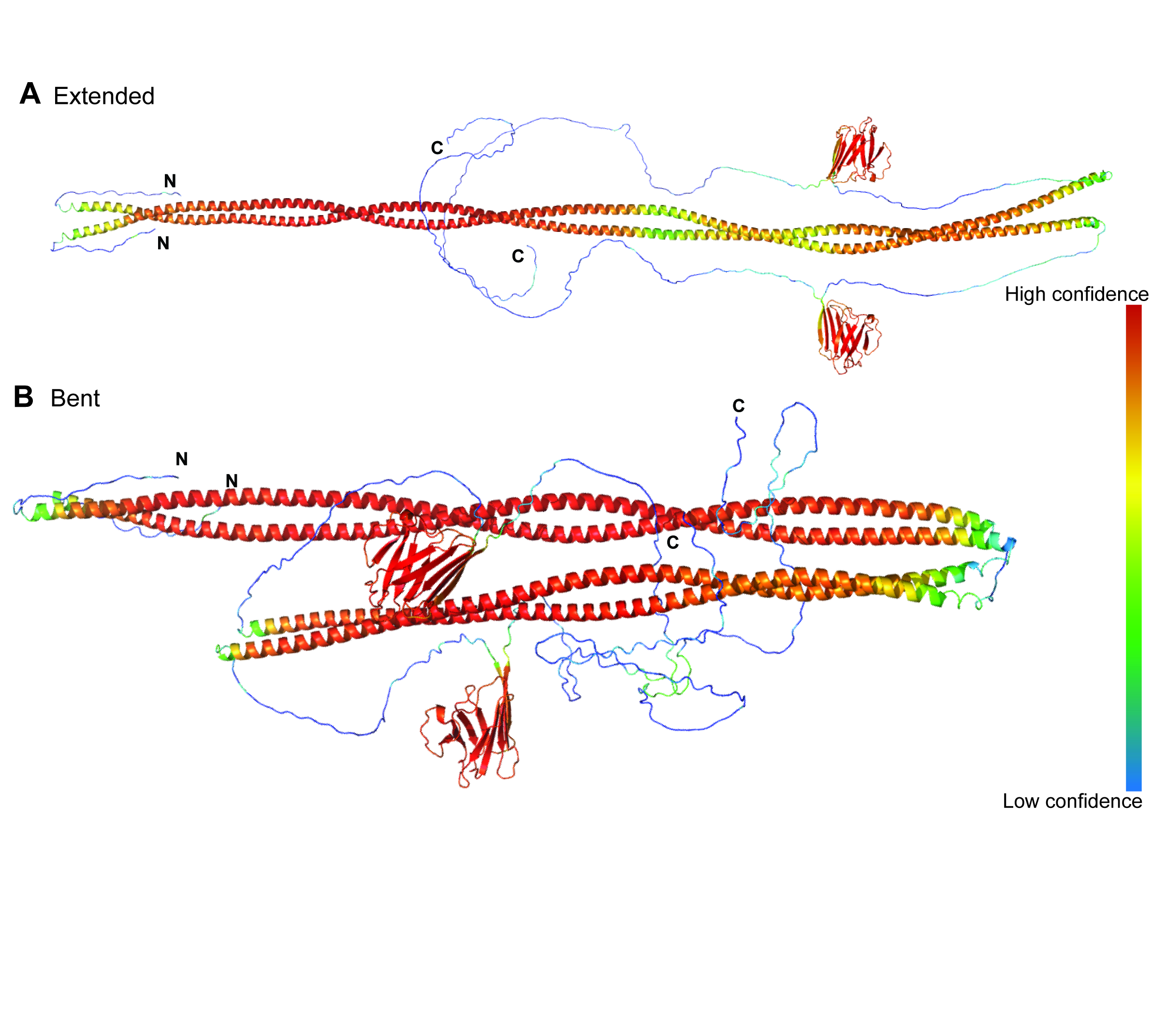

Supplement: Supplementary file 1 [file ijms-22-11226-s001.zip › Figure S3.tif]

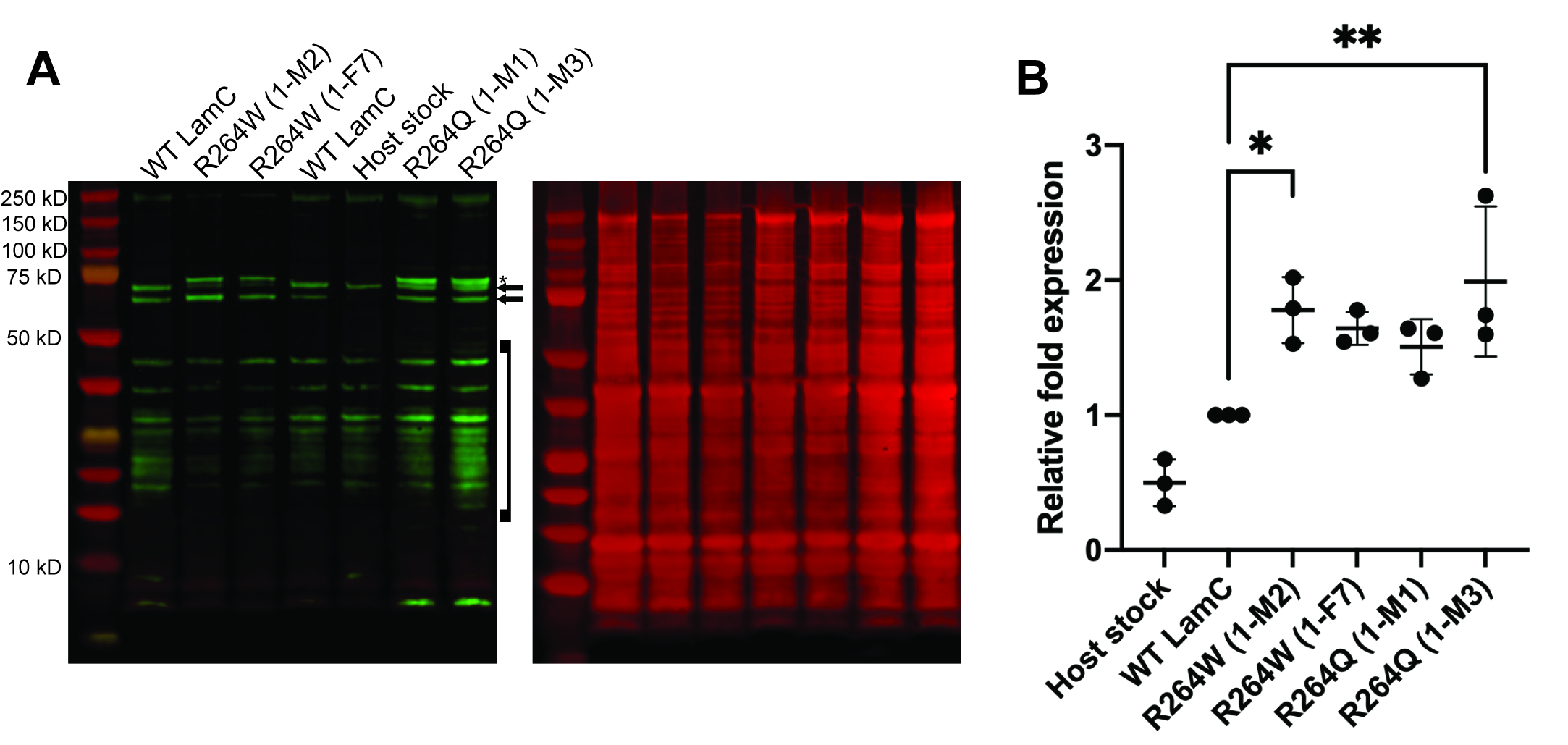

Supplement: Supplementary file 1 [file ijms-22-11226-s001.zip › Figure S4.tif]

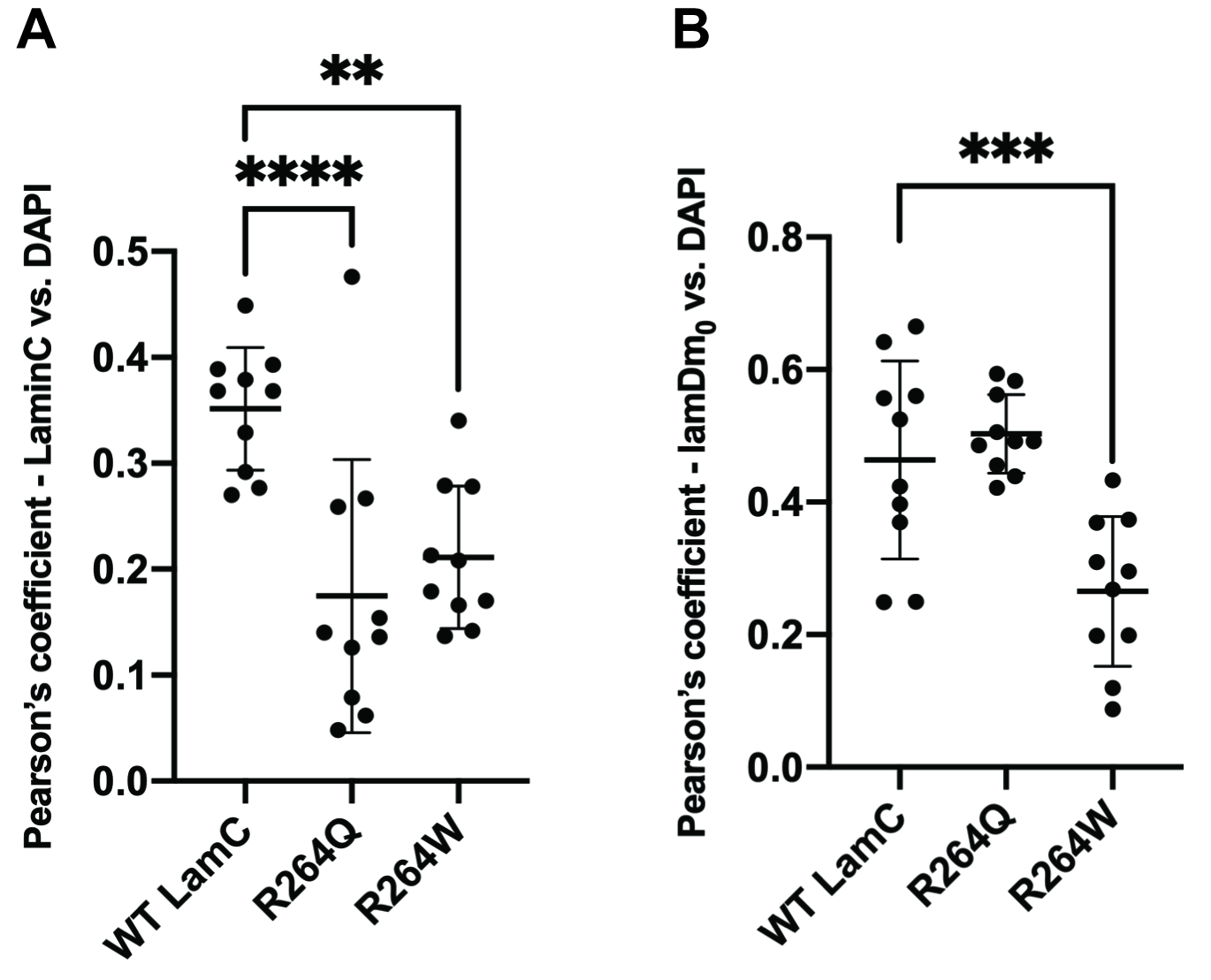

Supplement: Supplementary file 1 [file ijms-22-11226-s001.zip › Figure S5.tif]
